# Extracts and Gleanings

**Published:** 1873-08

**Authors:** 


### PART II.

### EXTRACTS AND GLEANINGS FROM OUR EXCHANGES.

### *MULTUM IN PARVO.*

A New Remedy for Colic.—About two years ago I had a mule suffering with a severe attack of colic, which threatened soon to terminate his existence. He would roll and tumble in the utmost agony, and continually threw himself against the ground with the greatest violence. I used all the ordinary remedies, resorted to in such cases—drenching with salt and soda, chloroform and laudanum, &c.,—without the least relief. I then put between one-half and two-thirds of a grain of atropia in half a pint of water, and drenched him with it; and in less than one minute he was on foot eating grass, with no sign of disease about him.

On the 26th of April, 1873, I was called to see Jack Thompson, (mulatto, aged thirty-three.) I found him complaining of severe pain in abdomen, which was swolen, tense, and so tender he could not bear the weight of my hand upon it. The pain seemed to be located chiefly around the umbilicus; tongue natural; no fever; bowels constipated. I do not think I ever saw any one suffer more severe pain in my life. The pain came in paroxysms, five or ten minutes apart. Treatment was morephine and laudanum, in large doses, cloroform, bromide of potash, valerian camphor, calomel, turpentine and oil, enemas, turpentine and camphor, stupes to bowels, with warm poultices. I do not say that these remedies were all used at once; but in the interval, from the 24th of April to the 5th of May, when he gradually got better of himself—my medicine not relieving the pain, nor doing any other material good, that I could see. On the 15th of the same month, I was again called to see Jack. I found him in just the same condition as described before, with pain equally as severe. For three days I used my old treatment without any result. At the end of that time, I concluded to try hypodermic injection of morphine and atropia. After preparing my solution, on attempting to brighten up my syringe, I broke off the needle. However, recollecting my experience with the "mule," I concluded to try the atropia alone. I placed about 1-20th or 1-30th of a grain upon the patient's tongue, and had him to swallow a little water. I remained about one hour afterwards, and, to my astonishment, "Jack" never had another pain. I then left three of the same doses, to be repeated every four hours. He had no more pain from the first dose, and went to plowing next day, feeling, as he expressed it, "a little heavy about the head and strange about the eyes." On the 31st of May and 12th of June, I was called upon to prescribe for Jack, in the same condition. One single dose of atropia relieved him completely, and instantly, each time. Now, Messrs. Editors, "one swallow does not make a summer," nor does one mule and one freedman establish the reputation of my remedy. But I wish my medical brethren to give it a trial, and let me hear from them through your valuable journal. It has certainly helped me out of the woods in two severe cases, and if it proves to be an instant relief for colic, it is easy to see how its usefulness may be extended in a certain class of spasmodic affections. Yours, &c.,

ROCKPORT, MISS. JAS. T. ALFORD, M.D.

QUININE AND UTERO-GESTATION.—About ten years ago I was interrograted, by a medical friend, as to my observation of the effects of quinine on enciente women; he adding, in the same connection, that, in his hands, there was a manifest tendency to precipitate premature delivery. Up to that time, there had been no such tendency of that drug, in my hands; and, from then till now, though I have given it freely in every period of utero-gestation, I have never seen the least untoward symptom follow its administration. I have been called to such cases, almost at term, when the friends really suspected the commencement of labor, and, in such cases, have given it freely, and the patient would have a good recovery from the fever, and go on to full term.

In the hands of the friend alluded to in the foregoing, *another drug* caused the trouble, as was ascertained by further inquiry. He was in the habit, *always*, of combining pulvis ipecac with the quinine. May not all the cases, where it has been accused of this evil, be due to some other drug? Unless there be an idiosychrasis, there can be no reasonable chance for quinine to produce such an evil. LOUIS B. BANCHELLE, M.D.

MIDVILLE, BURKE COUNTY, GA.

TO BEND GLASS TUBES.—Fill with sand and close the ends. The tube bends with heat, preserving the bore in perfect form. Lead tubes are filled with sand before bending, for the same purpose.—*Jour. Franklin Inst.*

A NEW REMEDY FOR SYPHILIS.—I believe, with Dr. Word of Decatur, "That there are thousands of useful and important facts now hidden from the profession, which should be made known." Therefore I send you a prescription obtained from an humble source, and one that has been in use, secretly, for many years, among the negroes of South-west Georgia and West Florida, in the treatment of that dreadful and loathsome disease that has shortened the existence of so many of the human family—viz., syphilis. My attention was called to it by an intelligent non-professional friend. I succeeded in getting the secret, and have used it in my practice for the last twelve years, with complete success.

℞. Bark of white ash root, commonly known as old man's gray beard, ℥xvi; prickly ash root—exanthoxylum fraxineum—℥vi; gun powder, grs. xlvii; pure gin, ℥iv; alumeris pulvis, ℈iv; water, O, vi.

Put the first two into the water and boil down to two pints. While hot, strain and add the other ingredient.

Sig.—A wineglass full three or four times per diem. Refrain from horseback exercise, salt diet, spirituous liquors, etc; regulate the bowels with any kind of gentle cathartic. If there be chancres wash them with the same.

Respectfully yours, B. I. A. CULL, M.D.
COLQUITT, MILLER COUNTY, GA.

PROPHYLAXIS IN SCARLATINA.—Dr. W. F. Dickenson, of Smithtown Branch, New York, writes to the *Medical and Surgical Reporter*, that during the recent prevalence of an epidemic of scarlet fever in his neighborhood, in which the prevailing type was simplex, yet a number being "anginose," and two exhibiting malignant qualities, the disease was arrested in its further spread, by sprinkling the floors with carbolic acid, and placing large plates containing the acid upon the tables in the different apartments. One family, having a number of children, in addition to the above measures, placed the acid in heaters and distributed its vapor through the house. For the children he also prescribed chlorate of potash in small doses, three times a day. In one family, who lived to one side, in a by-place, and was accidentally overlooked, the disease prevailed among all the children, and one had it in a malignant form. In this connection we call attention to the following:

Plugge has published in Pluger's *Archives*, a paper showing in a strong light the great power of carbolic acid as a disinfectant and a destroyer of infectious germs and organisms. The inquiries of Plugge have proven that carbolic acid not only modifies the putrefaction process, but absolutely destroys all the elements on which these processes depend. He found that vibriones, bacteria, monads, etc., were immediately destroyed by a solution of carbolic acid containing one to one and a half per cent. of the acid, and that a weak solution, though not sufficiently potent to utterly destroy them, arrested their further production and increase.

Infusions of meat, bread, milk, and all these combined were kept for a considerable period free from putrefaction by adding from 1-250 to 1-1000 of carbolic acid to them, and little or no development of these germs were known to occur in them. He further found that the acid as well prevented as arrested the alcoholic, butyric and peptonific fermentations. Four per cent. of the acid would arrest and disintegrate any fermentating process, and 1,1000 of it would prevent its continuance. Of late a very silly and unfounded condemnation of carbolic acid as a disinfectant has arisen from several pretentious quarters, but scientific inquiry shows this to be a captious outcry. Dr. Dickenson says he had no faith in his labors of love, to prevent the further spread of the disease, by disinfecting with carbolic acid the houses of the uninvaded families. However, let him and all others be "well rooted and ground," in the faith that carbolic acid is the best, safest and least expensive, good and practicable disinfectant the physician and hygienest have at their command, and that a liberal and wise use of it in times of need will always repay their confidence in its virtues as a disinfectant; ever recollecting that an ounce of prevention is worth *much more* than a pound of cure.—*Med. Archives.*

Quinine by the Rectum.—Sulphate of quinia is one of those agents which produce their proper effect through the rectum, particularly in children, in nearly the same quantity as when taken into the stomach. The *Detroit Review* remarks on this subject: "In our experience, quinine will do its work just as well when mixed with cocoa butter and pushed into the rectum, as when taken by the mouth. This can be done with less trouble and discomfort than attends the swallowing of a sugar-coated pill. The doctor, patients and attendants who have once tried this method of giving quinine to children and fastidious people, will be sure to remember and practice in similar cases. There is no way equal to this for administering narcotics when the stomach is weak or the patient unwilling to take medicine per mouth."

Electro-magnetic Paper.—If the extent of the employment of electricity as a therapeutic agent can be measured by the number of apparatus brought before the public from time to time, it certainly must be very widely used. The *Aerzt. Liter nt. Bllt.* speaks in the highest terms of Royer's electro-magnetic paper in the treatment of rheumatism, neuralgia, gout, etc. In point of constancy, utility and cheapness, it is stated to be unsurpassed.—*Medical Press and Circular.*

PHYTOLACCA DECANDRA IN THE TREATMENT OF INFLAMMATION OF THE MAMMARY GLANDS.—G. W. Biggers, M.D., of La Grande, Oregon, says in the *American Journal of Medical Sciences:* The following cases are stated as the *result of my experience* only with the remedy in question, and I trust that others may try it and report the result:

*Case I.*—Mrs. H., on third day after labor with her second child; mammæ commenced swelling, from an accumulation of milk. Did not see her until the symptoms were so urgent that there could be no mistake about the commencement of an abscess.

I pursued the antiphlogistic treatment, both general and local, until there was no promise of improvement; on the contrary, the case was continually getting worse. I then prescribed fluid ext. phytolacca decandra, gtts, xx, every three hours, in water. A very marked improvement took place in twelve hours, and in thirty-six hours the patient was well. There was also a suppression of the lochia, which was also reëstablished.

*Case II.*—Mrs. B., whose child died a few hours after its birth, was attacked, and after the secretion of milk took place, with inflammation of the mammary glands, from over-distension, and had the milk withdrawn very regularly, yet the case continued worse, threatening an abscess. I prescribed fluid ext. phytolocca decandra, gtts, xx, every three hours. Marked improvement in ten hours, and a complete recovery within thirty-six hours. There was also a suppression of lochia in this case, which was reëstablished with the cessation of the mammary inflammation.

*Case III.*—Mrs. G., at the fourth month of pregnancy, was attacked with inflammation of both mammæ, severe pain, swelling, and very great heat, with severe rigors, amounting to a distinct chill. I prescribed fluid ext. phytolacca decandra, gtts, xv, every three hours, in water. The symptoms all subsided, and the patient fully recovered within forty-eight hours, with no other treatment.

I have used the remedy above named in many other cases of mammary inflammation, and it has never yet failed in a single case.

The preparation used was Thayer's fluid extracts, for the reason that the plant does not grow in this State. A tincture from the green root would, I think, be more reliable.

BROMIDE OF POTASSIUM IN CHOLERA.—Dr. William Pepper, *Medical Times*, July 12th, 1873, recommends the use of bromide of potassium in the collapse of cholera. He advises it given in doses of forty-five grains in three ounces of water every twenty minutes, by mouth or injection. This drug, he thinks, has a wonderful power in quieting irritation of the sympathetic nerve, which irritation he regards as the source of the symptoms of relapse.

The Treatment of Boils.—Dr. J. D. McGaughey, of Wallingford, Conn., in a practical article on boils in the *Philadelphia Med. Times*, highly recommends sulphate of quinine for their treatment, and says:

I think the best plan to follow in giving quinine is—considering the aborting period to have been passed—to commence as soon as possible and make a firm impression on the system with five-grain doses repeated every four hours until the head rings, keeping the impression up moderately by smaller and oft-repeated doses until the boil has either suppurated or disappeared by resolution. A strict watch should be kept, so that the first pimple may be detected and incised; at the same time five or ten grains of quinine are given daily till all danger is past. No one need think he is going to be successful in circumscribing the trouble if he begins to give quinine just as suppuration is commencing. The great desideratum is to make a thorough impression on the system with the drug, keeping this impression up moderately with smaller doses, *for a considerable length of time*, according to the requirements of each individual case. According to my experience, arsenic—Donovan's solution, preferably—stands next to quinine. In concluding, I hope physicians will give quinine a thorough and judicious trial in furunculi, be they single, multiple, or in successive crops, as I think it is the only remedy that will circumscribe the limits and shorten the duration and prevent successive crops of those pestiferous sores.

Acute Articular Rheumatism.—When one of these patients is brought in with joints swollen and tender and painful, and motion about suspended, in short, with all the phenomena of an attack of acute articular rheumatism, he is immediately placed upon a treatment which consists in the administration of iodide of potash in fifteen grain doses every two hours, and sulphate of quinia gr. x, alternately, every two hours.

This is continued until the acute symptoms subside. It is expected that this will take place within fifty-six hours, and is discontinued at the end of this time in case the acute symptoms do not yield. In most cases the acute symptoms are completely subdued within twenty-four or forty-eight hours, and the patients feel comparatively comfortable. This, with a certain sense of propriety, might be regarded as heroic treatment, yet the results sanction and commend its adoption. The local treatment scarcely goes beyond covering the joints with cotton. Later in the treatment colchicum enters, and is regarded as a useful adjuvant to the salts which are ordinarily employed. It is said that the salts do much better when combined with colchicum, than when they are used alone.—*St. Luke's Hospital, N. Y.*

Hypodermic Treatment of Collapse.—Mr. Penfold reports a case of collapse, in the *Australian Medical Journal*, the result of severe purging. The patient was hardly able to articulate above a whisper; indeed, he was rapidly dying. Strong stimulants, mustard poultices, and the external application of heat, were tried without effect. "After waiting for an hour," Mr. Penfold says, "finding there was no improvement in his condition, and believing that in a few minutes he would be dead, and, in spite of his friends' remonstrances to 'let him die quiet,' I injected 20 minims of a mixture of the strongest solution of ammonia with three times its volume of water into the left radial vein and connective tissue (into the latter by accident from want of sufficient assistance.) In a few moments I thought the pulse was a little improved, and repeated the injection. After a short interval the man opened his eyes and tried to push away the syringe inserted in the vein with his right hand, and said 'Dash it, leave off,' in a moaning sort of way. From that instant he began to recover, and was able to take a little beef tea and brandy in the afternoon."

Hypodermic Injection of Sulphate of Morphia in Autumnal Catarrh.—Dr. Wm. Moss, of Chestnut Hill, Philadelphia, says, in *American Journal of Medical Sciences*, I first tried this remedy in my own case, three years ago, having previously had slight relief from the the internal use of morphia, and hoping only for a few hours' respite from the miseries of a violent attack of autumnal catarrh. The relief was immediate, and, to my surprise, lasting. I have since used it in a number of cases of autumnal and June catarrh with almost invariable success. It is sometimes necessary to repeat the injection one or more times during the period of susceptibility.

While far from claiming that this treatment is a specific, I am confident that it will bring a speedy relief to many who are unable to avail themselves of Dr. Wyman's pleasant remedy—a jaunt to the White Mountains.

Neuralgia.—One of the best methods of treating neuralgia is that recommended by Trosseau. A tight top thimble is filled with cotton wool, and a few drops of strong aqua ammoniæ dropped on it. The open mouth of the thimble is then applied over the seat of pain for a minute or two, until the skin is blistered. The skin is then rubbed off, and upon the denuded surface a small quantity of morphia (gr. ¼th) is applied. This affords almost instant relief. A second application of the morphia, if required, is to be preceded by first rubbing off the new formation that has sprung up over the former blistered surface.—*Canadian Pharma. Jour., May,* 1873.

EXTREME DEPRESSION AND PARALYSIS PRODUCED BY HYDRATE OF CHLORAL.—Henry J. Manning details (*Lancet*, May 17, 1873) two cases of monomania with exacerbations of excitement and obstinate sleeplessness, in which the administration of chloral was attended with very alarming symptions. In one case, an elderly man, who had taken five grains twice daily and thirty grains on alternate nights for about two months, became so weak and depressed that he was unable to make the slightest exertion, and suffered from partial loss of muscular power in the lower limbs.

In another case, ten grains twice daily, with forty grains on alternate nights, produced, in three weeks, the same condition, with cold surface, fluttering pulse, and complete paralysis of the lower limbs.

In neither case was there any loss of sensation or any hyperæsthesia.

Liq. strychniæ was prescribed, and in a few days both patients regained their usual condition.

VALENTINE'S PREPARATION OF MEAT JUICE.—An analysis, by Dr. W. H. Taylor, of Valentines preparation of meat juice, gave the following results:

Water, 61.12; organic substances, 27.90; inorganic substances, 10.98—100.00.

The organic substances include coagulated albumen (about one per cent. of the extract,) creatine, creatinine, and other constituents of flesh juice and blood. The inorganic salts consist of

Chloride of sodium, 1.42; sulphate of potassium, .52; phosphates of iron, calcium and magesium, 1.21; phosphates of potassium and sodium, 7.83.

It will be seen from this analysis that Valentine's preparation differs essentially from Liebig's extract of meat, in containing a large proportion of those nitrogenized principles to which meat owes its chief nutritive value.

MOSQUITO NETTING AS A SURGICAL DRESSING.—In all those cases where it is desirable to keep up support and pressure, and at the same time permit the free escape of all discharges from the wound, or ulcer, or whatever it may be, the ordinary mosquito netting used for a bandage meets all the indications. Bundling dressings are avoided in this way, the parts are kept cool, the discharge goes on unrestrained, and at the same time support is maintained. If the discharge is considerable, a pad of oakum may be placed beneath the parts to secure the discharge, thus insuring perfect cleanliness. This netting serves an admirable purpose in dressing large abscesses; for instance, when compression and free discharge are to be associated.—*St. Luke's Hospital, N. Y.*

Bromide of Potassium and the Diseases of Dentition.—Dr. C. G. Polk, M.D., of Philadelphia, (*Medical and Surgical Reporter*) says: As bromide of potassium has been applied to nearly every disease to which flesh is heir, anæmia and gastritis perhaps not excepted, I supposed, until recently, that the value of this agent in the disorders of dentition was well understood, and that it was frequently nsed. Finding the application of it to that class of disorders quite a novelty to many, I will give my experience, hoping to awaken attention to it from those possessing larger fields of observation, to verify or disprove my conclusions.

I have used bromide of potassium in about one hundred cases of infantile diseases, embracing those of diarrhœa, pulmonary congestion, and cerebral congestion, arising from dental irritation. I have seen the diarrhœa which had defied chalk, acetate of lead, calomel, catechu, opium, the sulphites, and pepsin, yield in forty-eight hours to this bromide. I have seen persistent vomiting, which is often, in such cases, nought else but reflex action from the brain, cease after a full dose of this agent. I have seen the hot head, with gritting of the teeth, presagers of convulsions, yield to a few doses. I have seen pulmonary congestion, dependent on the same cause, yield in a few days.

The most marked case I recollect was in a daughter of Mr. Pickett, of Frankford Arsenal. I had diarrhœa and both cerebral and pulmonary congestion to contend with, and at the time I resorted to the bromide of potassium, the case seemed hopeless; under its influence she made a rapid recovery.

The dose must be adapted to each case.

Night Sweats of Phthisis.—A remedy commonly employed for the relief of this symptom is: Fld. Ext. Ergot in drachm doses at night. In some cases the patients vomit the remedy, but it is said to work exceedingly well in a large majority of cases.

Hydrate of chloral, given in grs. xx. doses about two hours before the time for the sweating to commence, is another plan.

Another method suggested, is to awaken the patient a little before the hour at which the sweating commences, have him wash himself and take a little lunch.—*St. Luke's Hospital, N. Y.*

Chronic Diarrhœa.—Rayer (*Union Medicale*, No. 73,) recommends the following combination in the treatment of this complaint:

℞. Bismuthi subnitratis, ʒi; cinchonæ flavæe, pulv. ʒss; carbonis ligni, ʒi; misce et in chartulas XX divide.

S. One powder two or three times daily, in the intervals between meals.

Calabar Bean in Constipation and Hemicrania.—Dr. Subbotin (*Deutsch. Archiv. Klin. Med.,*) advises this medicine in constipation with atony of the intestines. The following is his formula—Extract of calabar bean, 20 centigrammes (about four grains;) pure glycerine, 8 grammes (2 drachms;) dissolve, and take four drops four times a day.

The *Morgagni* tells us that Dr. Green uses extract of cannabis indica in treating cases of hemicrania. He prefers the extract to the tincture, both on account of the bad taste of the latter, and because he believes it to be less active, and more easily changed in composition. The alcoholic extract is the better preparation, if it be perfectly pure and recently prepared. The doses vary according to the intensity of the disease, a third of a grain every morning, or morning and evening, is enough. In anæmic persons cod oil may be given at the same time.—*Doctor.*

Carbolic Acid as a Local Anæsthesia in Boils, Whitlows, etc.—Dr. J. N. Taylor (*Nashville Medical Journal*) writes:

I have frequently used carbolic acid in minor surgery, but not until recently to my great satisfaction.

About the middle of November of last year, I was the subject of a very large and painful boil, on the ulna side of the forearm. I saturated a piece of worn linen in an aqueous solution of carbolic acid, and applied until all sensibility of the part had ceased; then, with a piece of ordinary writing-paper, twisted to a sharp point, and dipped into a solution of the acid (full strength,) marked out the line of the desired incision; then, with an ordinary scalpel, I made a free incision, fully an inch in length, without any pain whatever.

I greatly prefer the application of the acid, thus used, to the freezing process.

Strychnia in Amaurosis.—Dr. J. J. Chisolm, in the *New York Medical Journal,* February, 1873, reports a case of amaurosis in which, beginning at the one-thirtieth of a grain of strychnia he gradually increased until one-fifth of a grain was reached. This great increase of the dose was made within two weeks. For a time improvement was rapid, but it then ceased. The dose was again increased, and the increase continued till seventeen-thirtieths of a grain of strychnia was taken daily. With this dose he continued to improve for eleven weeks, when he was able to resume business. This case is selected from a large number, to illustrate the reason why strichnia fails as a nerve stimuli in so many instances. The doses given are not large enough to produce the medicinal effects of the drug.—*Review of Medicine and Pharmacy.*

Gelseminum in Gynæcology.—Dr. H. V. Ferrel, (*Clinic*) says: In preparing for the application of arg. nit. in endometritis, it was thought advisable to employ gelseminum to relieve the excessive nervous irritability which rendered manipulation of the uterus next to impossible. I gave three 10 gtt. doses fl. ext. gelseminum four hours apart, and found the nervous excitability gone, and the os uteri, which before was contracted and rigid, already dilated.

Called Jan., to see Mrs. ——, for suppression of lochia as alleged by two physicians already in attendance. Patient had been confined eight days previous, attended by a midwife, who, in attempting to "clear her," had torn away the cord and part of the placenta, leaving the remainder. Present condition of patient alarming.

On making digital examination I find the os so contracted as to barely admit the tip of the index finger. I gave gelseminum and belladonna. Twelve hours after, found the patient easy; os dilated so as to admit two forefingers which detached and brought away the retained portion of the placenta with membranes. Patient recovered without further trouble.

If this is one of the effects of gelseminum, either alone or combined with belladonna, its utility becomes at once apparent to gynæcologists.

Erysipelas.—The *N. Y. Medical Record* gives the following as the treatment at Charity Hospital: The only successful method of treatment which has been employed for arresting the spread of the disease, is the formation of a boundary-line by means of a blister. For this purpose, the vesicating collodion is ordinarily used. If erysipelas attacks a limb, encircle it with a blister about one-half or three-quarters of an inch wide, at a free point above the part affected.

A very common, and most excellent, application for the erysipelas, is an ointment composed of—℞. Sulphate of iron, ʒi; sapo or unguentum ceræ, ℥i. M.

In this way the iron can be used, and the stains avoided, which are so annoying when employed in solution.

Burns.—Gunpowder burns are occasionally brought in from among the quarrymen, and the application used for this class of injuries is, bichloride of mercury, one grain to the ounce of water, with the addition of one drachm of tr. benzoin. This is an old prescription here, and is supposed to be especially serviceable in connection with gunpowder burns.

Oxide of zinc ointment has the practical recommendation of an old member of the fire department.—

Hydrate of Chloral in Sclerosis of the Middle Ear ("Dry Catarrh").—These cases Prof. Lucæ, of Berlin, characterizes as being without any essential structural changes in the membrana tympani and in which the air douche has no effect. A few drops of a solution of one part of chloral to thirty parts of water are injected in the usual manner into the ear. The immediate severe pain disappears, as a rule, in a few moments. The redness excited in the membrane by the injection, varies in different cases. If on the day following the injection, the former dry, *hard* sound on inflation is changed into a soft one, the prognosis is less unfavorable than in those cases where neither optical (hyperæmia of the membrane) nor ausculatory changes are observed. The operation of the injection is regulated by the reaction, ordinarily twice a week, Improvement, when it takes place, is observed after the first injections. Deterioration never follows. Likewise, no active inflammation in the tympanic cavity. In 100 cases decided improvement in the hearing took place in 11, and slight improvement in 25. The tinnitus for the most remained unchanged. In 5 cases the improvement in the hearing still continued after a year, in 1 even *after three years.—Arch. f. Ohrenhlkd.—Clinic.*

Hxpodermic Injection of Quinia in Heat Apoplexy.—Mr. A. R. Hall, Assisrant Surgery Royal Artillery, states (*Indian Med. Gaz.*, Sept 2, 1872): "I think that all the well-known symptoms of heat apoplexy are produced by *intense nervous exhaustion*, and that it is a pathological condition closely allied to the *secondary fever* of cholera. I have seen the utmost benefit result from the hypodermic injection of quinia in insolation, where actually moribund patients have been saved by it. I would employ the same remedy in the *secondary fever* of cholera. In the number of the *Indian Annals of Medical Science* for March, 1870, I brought forward the theory that in the *collapse* of cholera there is very great *irritation* of the sympathetic nervous system. I recommend for that condition the hypodermic injection of pure sedatives." Mr. H. thinks that in the hypodermic injection of quinia we have the remedy for heat apoplexy.—*American Journal of Medical Sciences.*

The External Application of Veratrum-Viride.—Dr. Anson Ford, of Buttle City, Montana (*Jour. Gynæcological Soc.,*) writes, that in the absence of all the remedies usually exhibited, he applied to *a supposed cancer of the left breast,* Norwood's tincture of veratrum-viride, and was astonished at its immediate and almost magical effect. The induration of the gland and the excruciating pain disappeared under this treatment. A cloth was kept over the gland, saturated with the solution, and over all oil silk, to prevent evaporation. Was it cancer?

MONTI ON CONSTIPATION IN CHILDREN.—Dr. Alois Monti (in the *Wien. Med. Presse*, xii. 26–28, 1872) contributes an exhaustive paper on this subject. He thinks it due to the defective development of the muscular tissue of the intestines, and to the peculiar form of nourishment at this period. He sums up the various causes: 1. Mechanical impediment, as in congenital deformity, imperforate anus, invagination, hernia, etc.; 2. Defective nourishment as from congenital defects of the lip and throat, too little secretion of milk, etc.; 3. Faulty nourishment, as from excess of casein or defect of fatty matters in the milk, bringing up by hand, starchy food, etc.; 4. Deficiency or diminution of the peristaltic movements of the intestines, atrophy, etc.; 5. Diminution of the intestinal secretion, as in long-continued diseases in consequence of anemia. Constipation is further a symptom of diseases of the brain and spinal cord; also a consequence of deficiency of drinks, of the use of astringents, presence of ascarides, fruit-stones, etc.; and, in older children, it arises in consequence of deficient bodily exercise. The cause suggests the remedy—cod-liver oil and enemata of cold water, mineral waters, manna, etc.

ACTÆA RACEMOSA.—Dr. F. H. Bailey uses the black cohosh in acute rheumatism, combined with alkaline salts, ipecac, and sanguinaria canadensis. In chronic rheumatism, with tonics and tonic alteratives. In chronic conditions of the liver, where there is dull aching pain in the organ and tip of shoulder, he uses it freely. The following is a favorite formulæ: ℞. Tr. actæa racemosa, ℥ii; iodide potass., ʒii; syrp. ipecac, ℥j; tr. sang. canaden. ℥ss. M. S. Teaspoonful three times daily. In rheumatism he uses a similar mixture, giving less of the actæa and more of the ipecac. He frequently combines tr. cinchona or sulph. quinine with it.

In a case of cholera he gave the following: ℞. Fluid extract valerian, fluid extract racemosa, aa. ℥j.; syrp. iodidi ferri, ℥ss; sulph. quiniæ, ℈j. M. S. Teaspoonful three times daily.

A NEW USE FOR OLD STOCKINGS.—A plan of putting up simple fractures of the leg, which is not, we believe, generally known, and which country surgeons may sometimes find convenient, has lately been practiced at University College Hospital. The broken limb is first bandaged with an ordinary roller; this is well coated with the gum and chalk mixture; a stocking is slipped on over this and similarly coated; another stocking is put on over this; and a final layer of gum and chalk over all. Thus, for a case of transverse fractute with little displacement, or of fracture of one bone, two or three stockings and a little starch or plaster of Paris supply a very neat and serviceable splint.—*British Medical Journal, May*, 1873.

Small-Pox Keratitis.—Dr. A. D. Williams, St. Louis, Mo., (*Med. Archives*), combines atropine with carbolic acid in the treatment of small-pox keratitis—the carbolic acid aiding greatly the effect of the atropine. The proportions are as follows: ℞. Atropine sulph., gr. iv.; acid. carbolic, gr. v.; aquæ destilat', ℥j. Mix. To be dropped in the eyes three to five times a day, according to severiiy of the keratitis; two or three times at night, if there is much pain. In addition to this treatment, in cases of deep ulceration of the cornea, where the healing process is slow, a very strong solution of the acid (15 to 30 grs. to the ounce) may be applied directly to the ulcer once a day. First cleanse the ulcer well, and dip the end of a small probe into the solution and touch the small drop that sticks to it directly to the bottom of the ulcer. This will spread over the ulcerated surface and turn it instantly white. It smarts sharply for a moment. Care should always be taken to confine this strong solution to the abraded surface of the cornea.

Sulphuric Acid as a Prophylactic in Cholera.—Dr. R. G. Curtis, in *Medical Times*, July 12th, 1873, advises the use of sulphuric acid as a prophylactic in cholera. His views are based upon observations made in the Philadelphia Hospital, during 1865 and 1866. He gave to all the inmates twenty drops of dilute sulphuric acid in four ounces of sweetened water. Each day, for six days before the use of the acid, there occurred an average of four new cases.

Four new cases occurred during the first twelve hours of administering the acid. After this, for one week, there were no new cases. Then owing to lack of materials, the medicine could not be given for two days. At the end of this period two cases occurred. Then the acid was renewed, and continued for some time, but no more cases occurred. For various reasons, the doctor thinks that these facts show that sulphuric acid did prevent the spread of cholera.—*Detroit Review of Medicine and Pharmacy.*

Radical Cure of Fistula Ani Without the Use of the Knife.—This proceeding, advocated by Dr. Lute, of America, consists in the use of an injection of a solution of iodine in ether. The ethereal tincture is more exciting than the alcoholic tincture, and the ether, evaporating rapidly, leaves the walls of the fistula in contact with the pure anodine. There is scarcely any reaction, and the patient need not stay in bed.—*London Lancet.—St. Louis Med. and Surg. Journal, June* 73.

The bitter taste of quinine, colocynth, aloes, quassia, and of other bitter medicines, may almost instantaneously be removed by chewing a small piece of liquorice root, says *La Tribune Medicale.*

Eucalyptus as an Antiseptic.—W. Gimbert says: "Phenic (carbolic) acid is an acid antiseptic—difficult, dangerous to manage therapeutically; eucalyptol, on the contrary, is not acid, and may be administered in large doses to patients without producing accidents." Eucalyptol is then recommended for poorly-nourished, hospital patients suffering from gangrene or fœtid suppurations of any kind. Mr. Gimbert proposes to stop anything like the spontaneous generation of vibrions bacterians *et id omnes genus*, by the application of a sufficient quantity of his new antiseptic. It is given internally as an antiseptic in cases of typhus, dysentery, ulcer of the stomach, septicæmia, pyæmia, scarlatina, and diptheria; also in fœtid bronchitis and gangrene of the lungs; in fact, in any case when there is a decided tendency towards the formation of pus. Eucalyptol is eliminated much more rapidly than any mineral antiseptic, and can be given while the patient is feverish, as it acts as a refrigerent.

Sponge-tent in Epistaxis.—Dr. James Young (*British Medical Journal*, May 17, 1873) recommends the use of sponge-tent in cases of bleeding from the nose, and gives the following method of preparation: "Have a long piece of fine sponge, dipped in a solution of gum, compressed with twine, dried; and, after the twine has been unrolled, the sponge is thickly coated over with white wax. This is easily passed along the floor of the nostril, leaving a piece of red tape for extraction. The tent may remain for six hours, and must be gently rotated before extraction, to prevent fresh hemorrhage."

Use of Placental Forceps through the Speculum.—Dr. H. Seaman, in the *Buffalo Med. and Surg. Jour.*, (April, 1873,) recommends the application of the placental forceps through the speculum. By this device he could readily see the part to be removed, grasp, and bring it away. Young practitioners, at least, may profit from this hint, and be able to remove the placenta safely and certainly. Further, in view of the cases recently reported, in which death followed the introduction of a hand into the uterus for this purpose, we think the suggestion worthy the attention of even the most experienced obstetrician.

Arnica Liniment.—Tincture arnica, ℥i; soap liniment, ℥iii. Mix. This is good; but the following lotion is generally preferred:

℞. Tincture arnica, ʒi; water, ℥ij; solution of subacetate of lead, ʒij. Mix and filter through paper. This does not stain the skin.

PERSISTENT VOMITING is treated at Charity Hospital (*N. Y. Medical Record*) as follows: There was a case of persistent vomiting in connection with Bright's Disease. The patient was a female, and this was the second attack she had suffered. A number of months previous, in her first attack, the vomiting was sufficiently persistent to reject all remedial agents employed for its relief, and it was supposed that the patient must then and there die. She was placed, however, upon treatment by the use of raw beef, or so nearly raw that it could hardly be said to be an infringement upon the proper use of terms to call it raw, and she began immediately to improve. Her recovery was complete so far as the vomiting was concerned. Her second attack had come on only a few days previous to my visit, and she was placed upon the beef treatment at once. The result was equally satisfactory with the first, and the patient was now able to take iced milk with her beef, and was feeling very comfortable. The beef, raw and seasoned with a little salt and pepper, or cooked in the slightest degree over coals and seasoned in the same way, is taken in quantities averaging about one ounce every three hours. It was cut in small pieces, set by the bedside, and the patient administered it "piecemeal."

CHILBLAINS.—F. Phien (*British. Med. Jour.*) recommends the following, remarking that four or five applications cure: About one ounce of tannin is dissolved in half a pint of water; seventy-four grains of iodine are dissolved in an ounce and three-fourths of spirits of wine; the two solutions are then mixed; and enough water is added to make up the whole to two and a half pints. The remedy is applied once daily—the best time being before going to bed. The mixture is gently warmed over a very slow fire; the affected part (*e. g.*, the hand) is dipped in it while still cold, and held there until the liquid, on being stirred, feels comfortably hot. The vessel is then removed from the fire, and the hand is dried over it, without gloves. The vessel used must be of earthen ware, or porcelain, not of metal. Care should be taken not to use too great a quantity of iodine, especially when abrasions are present.

NÆVI CURED BY MONSEL'S SOLUTION APPLIED EXTERNALLY.—Dr. Geiger, in the *American Practitioner,* (April, 1873,) recommends the external application of equal parts of liq. ferri persulph. and glycerine to the surface of nævi and a little of the adjacent skin. In two cases in which the applications were made twice daily, the nævi disappeared in less than a month.

THE so-called "pain-killers" are usually made of equal parts of liquor ammonia, sulphuric ether and alcohol. The oils of peppermint, sassafras and wintergreen will make it all the better.

DISPLACEMENT OF THE UTERUS TREATED BY ELECTRICITY. In an article upon this subject, (*Medical Record*, April 15, 1873), Dr. E. C. Mann presents good grounds for believing that many cases of displaced uteri can be radically cured by the proper electrical treatment. He reports four cases of prolapsus uteri of the first degree entirely cured by such treatment. The causes of the prolapse were a loss of elasticity of the uterine ligaments, atony of the vaginal walls and sphincter vaginæ. The negative electrode was placed in the uterus, and the positive externally over the uterus and its appendages, and the faradic current employed. He gives in detail the treatment of an old case of prolapsus, as illustrating the effects of electrical currents to restore tone to the general system, and especially to the tissues in and about the pelvis. He thinks it well to combine the galvanic and faradic currents. In all cases nutrition is improved, and many nervous symptoms connected with displacements entirely relieved.—*Detroit Review of Medicine.*

NEURALGIA AND SCIATICA CURED BY TURPENTINE—TURPENTINE PEARLS.—Dr. Martinet, in a memoir presented by him to the Faculty of Medicine, affirms that he has cured fifty-eight cases of neuralgia and sciatica out of seventy by the use of the essence of turpentine. The great efficacy of this agent is not to be doubted in the above-named affections; and what is remarkable, the benefit is almost always felt from the first doses. But this medicine has such a disagreeable odor, such a harsh and pungent taste, that it is quite impossible to take it pure. Dr. Clertan has contrived to inclose it in very thin and transparent gelatine, in the form of little globules, of the size of peas, to which he has given the name of turpentine pearls. Dr. Clertan's "turpentine pearls" are as readily swallowed in a little water as pills. This agreeable form has made the essence of turpentine quite popular; and now there is not a physician (so says the announcement) in France who does not resort to turpentine pearls in the treatment of neuralgia and sciatica. Trousseau always prescribes turpentine in this form.

ULCERS.—A dressing which is said to serve a most admirable purpose for any ulcerated surface which may need a soothing and slightly stimulating application, is the ceratum resinæ et bals. Peruvian. It is usually employed in the proportion of one part of balsam to four of ceratum.

HOPS AS AN EXTERNAL ANODYNE.—Enclose the hops in a bag, and subject it to the steam of boiling water, and apply as warm as can be borne. Dampening the hops *slightly* with strong vinegar before steaming, increases their anodyne virtues.

Salicin in Obstinate Diarrhœa.—Dr. I. B. Mattison, of Chester, New Jersey, says, in the *Phil. Med. and Surg. Reporter*, that an assertion that the majority of practitioners, during an active professional life, meet with one or more cases of diarrhœa which prove utterly rebellious to ordinary treatment, will, we presume, pass unchallenged. After an experience limited to a few years, we have the record of several such instances, and the success in our hands attending the use of salicin has been so marked and gratifying that we are induced to place it before the profession, for the benefit of those who may not as yet have given this remedy a trial under similar circumstances. We administer in powder or pilular form, to children preferably the former, in any appropriate vehicle, in doses, under two years of age, of one-half grain every four hours, and to adults after the following formula: ℞. Salicin, ʒj.; fiat pill, No. 24. Sig. Two pills every four hours. Its employment is followed after a short time, by a decrease in the frequency of the evacuations, a return to their normal color and consistence, and subsequent restoration to entire health.

Rules for Giving Belladonna in Opium Poisoning.—Dr. Mary C. Putnam, in the *Medical Record*, June 2, 1873, gives the following rules for the use of belladonna in cases of opium poisoning: 1. It should not be given as a prophylactic, but only to combat conditions already existing, either of restlessness, nausea and vomiting, or somnolence, stupor or coma. 2. It should not be given in large doses, but in small ones, (tinct. ♏ xv.) frequently repeated. 3. It is safe to continue the administration, so long as the pupils are not dilated, nor the pulse accelerated. If dilatation has taken place, yet the iris remains motionless, if the pulse has become weak and rapid, and the coma still continues unabated, further use of the atropine would increase the mischief. 4. The use of adjuvants, as emetics, coffee and electricity, is to be recommended as much in the belladonna treatment as in any other. The prevision of the physiological effects to be expected from belladonna, enables us generally to analyze its influence, even when a complete medication has been employed.

Chanchroids.—A goodly number heal rapidly when dressed with pure iodoform. Occasionally a rebellious one needs to be once thoroughly cauterized, and after this will heal kindly by the use of the iodoform.

Bright's Disease.—The general principles of treatment embrace the administration of tonics and diuretics. The best tonics are, quinine and tr. chloride of iron; the best diuretic is thought to be the infusion of digitalis.—*St. Luke's Hospital, N. Y.*

Podophyllin.—Dr. Constantin Paul lately read a paper on this drug at the Société de Therapeutique, the publication of which has just been concluded in the *Gaeette Médicale de Paris*. He considers this remedy one of the most reliable in habitual constipation. He began by combining it with belladonna, as advised by Trousseau and others. When belladonna did not agree, he substituted hyoscyamus. He has now discarded all adjuvants, and, with a smile at the polypharmacy so common on our side of the channel, he recommends a small dose of podophyllin made into a pill with honey, to be taken every night. In the constipation of pregnancy and uterine disease, he has found it the best remedy, producing a single evacuation each morning. Should there be more effect after a few days, he omits the dose for a night or two.—*Doctor*.

Erysipelas.—For a local application, the liq. plumbi et opii is used, and for internal administration, quinine and iron; and the quinine is thought to be especially serviceable. It may have been noticed that the administration of quinine and iron in some of the hospitals is not so steadily adhered to as has been wont to be in the case with the profession in general. Of their value in this disease, it will be difficult to convince the profession, notwithstanding some of the cases which are treated without the administration of these remedies seem to do equally as well as those which receive it.—*Medical Record*.

Formula for Hysteria.—The following is a valuable antispasmodic: ℞. Tincture of castor, tincture of assafœtida, of each, ℥iss.; camphor-water, ℥i.; aromatic spirits of ammonia, ℥ss.; syrup of acacia, ℥iss. Mix. S. Teaspoonful as occasion demands.

Cramps Attending Pregnancy.—The following has been used with benefit: ℞. Oil of amber, fʒss.; liquor potass., fʒj.; camphorated tincture of opium, f℥ss.; peppermint water to make ℥vj. Mix, and take two tablespoonsful at bed-time.

Pruritus Vulvæ.—Take of bichloride of mercury, one part; alum, twenty parts; starch, one hundred parts; water, twenty-five hundred parts. Mix. S. Apply freely to the part.—*Revue de Thérap.*

Vermifuge Syrup.—Extract spigelia maryl., fl.℥viii.; syrup sennæ compos. (Lond. Ph.), lbs. ii. Mix while hot, and evaporate to a proper consistence. Dose, a small teaspoonful for a child one year old.

Bromide Potass. is given in large doses, with satisfactory results, in the second stage of Bright's Disease.

ATROPIA SUBCUTANEOUSLY IN CHOLERA.—Dr. Hodgen mentioned (Proceedings St. Louis Medical Society, June 7, 1873) a plan of treatment pursued by him in a few cases at the close of the epidemic of 1866, with results considered sufficiently encouraging to merit further trial. In the *stage of collapse*, the Doctor injected subcutaneously from a sixtieth to a thirtieth of a grain of sulphate of atropia, and injected freely into the bowels warm water with a little salt in it; reaction and convalescence followed in over half the cases without fever. The Doctor also referred to the importance of promoting the action of the kidneys as early as possible, by diuretics and warm poultice or fomentation over the kidneys, not to eliminate the cholera poison, but to obviate uræmia.—*St. Louis Medical Journal.*

TREATMENT OF DIABETES MELLITUS.—Schultzen advises, in cases of saccharine diabetes, the following prescription: Very pure glycerin, 20 to 30 grammes; water, 700 grammes; citric acid, or tartaric acid, 5 grammes. To be drunk in the course of the day (about a fluid ounce of glycerin in the twenty-four hours). This dose may be kept up for months without any injury being done; but if much more glycerin be given, diarrhœa may arise.—*Doctor.*

ATROPIZED CASTOR OIL IN CORNEAL DISEASE.—Dr. Owen, (*British Medical Journal*), recommends the above combination in the treatment of irritable ulcer of the cornea, abrasion of the epithelium, etc. In these cases, it is desirable to fill the inequalities of the surface, and so to reduce the irritation caused by the movements of the eye to a minimum. He adds from one to four grains of atropine to each ounce of castor oil.—*Detroit Rev. Med.*

SUBACUTE PLEURISY.—Patients who present their credentials and are booked, "effusion into the pleural cavity—large quantity," are tapped at once, and placed upon tonics, quinine and iron. Diuretics are not used with any great degree of confidence. When the effusion is moderate, tonics alone are used.—*Medical Record.*

FOR CATARRH.—Pure carbolic acid, five parts; alcohol, fifteen parts; strong liquor ammonia (Sp. 8, 960), five parts; distilled water, ten parts. Keep in a black, glass-stopped bottle. It should be prepared in small quantities only. A few drops should be poured on filter paper, and inhaled every two hours.

STOKES' LINIMENT.—℞. Oil of turpentine, ℥iij.; strong acetic acid, ℥ss.; rose-water, ℥iij.; oil of lemon, ʒi.; yolk of one egg.

JUNIPER TAR SOAP.—Oil of juniper, ℥i.; soft soap, ℥i.; alcohol, ℥i. Mix. Useful in some obstinate skin diseases.

Action of Ergot.—About this much-discussed subject, there is a paper in the *Proceedings of the Dublin Obstetrical Society*, by Dr. Denham. He concludes: Ergot produces only nausea and loss of appetite when given during pregnancy at any other time than the full period; it does not produce an injurious effect on the fœtus in utero; when abortion has commenced, ergot hastens and assists it. In the second stage of labor, it is often beneficial; but if the labor be not soon completed, it is very dangerous to the child by mechanically obstructing the circulation, and by the powerful and continuous contraction exerted on the child.

Ice in the Treatment of Facial Neuralgia.—M. Wenteritz reports a case of very intense facial neuralgia which resisted every mode of treatment, that finally yielded to the application of ice along the affected side of the face, laid on at periods of five minutes. The pain disappeared in twelve hours, and after an interval, now of ten months, has not returned.—*Med. Press.*

Etherized Cod-Liver Oil.—Ether given in conjunction with cod-liver oil is believed to render it more digestible by the pancreatic fluid. The following formula is recommended by Dr. B. W. Foster: ℞. Cod-liver oil, ℥i.; *pure* ether, forty to sixty minims. Dose, a tablespoonful, to be increased as the case may require.

Treatment of Croup.—Dr. Welsh recommends the use of iodine, given internally, in the treatment of croup, and relates, in confirmation, a successful case treated by one or two-drop doses of the tincture every half hour.—*Doctor.*

Tincture Iodine, Colorless.—Tr. iodine, ℥i.; glycerin (pure), ℥i.; sulphite of soda, ʒi. Add the glycerin to the sulphite carefully; then pour in the tincture; triturate gently until the union is complete.

Laxative Powder of Jeannel.—℞. Rochelle salts, fifty parts; white sugar, one hundred parts; bicarbonate of soda, twenty-two parts; tartaric acid, powdered, twenty parts; oil of lemon, q. s. Mix. Dose, a teaspoonful in sweetened water.

For Chilblains.—℞. Muriate ammonia, ℥ss.; aquæ dest., ℥iv. Mix, and apply night and morning. This gives positive relief.

Mucilage for Office Use.—Gum Arabic, ℥i.; glycerin, ʒij.; boiling water, ℥iij. Dissolve.

Neuralgia—Gray's Powder.—℞. Tincture of aconite, chloroform, each, five parts; lard, twenty parts. Mix.

